# CAR T-Cell Therapy for B-Cell non-Hodgkin Lymphoma and Chronic Lymphocytic Leukemia: Clinical Trials and Real-World Experiences

**DOI:** 10.3389/fonc.2020.00849

**Published:** 2020-05-27

**Authors:** Candida Vitale, Paolo Strati

**Affiliations:** ^1^University Division of Hematology, A.O.U. Città della Salute e della Scienza di Torino and Department of Molecular Biotechnology and Health Sciences, University of Torino, Turin, Italy; ^2^Department of Lymphoma and Myeloma, The University of Texas MD Anderson Cancer Center, Houston, TX, United States

**Keywords:** CAR T cells, indolent B-cell lymphomas, aggressive B-cell lymphomas, chronic lymphocytic leukemia, clinical trials, real-world experience

## Abstract

Chimeric antigen receptor-modified (CAR) T cells targeting CD19 have revolutionized the treatment of relapsed or refractory aggressive B-cell lymphomas, and their use has increased the cure rate for these cancers from 10 to 40%. Two second-generation anti-CD19 CAR T-cell products, axicabtagene ciloleucel and tisagenlecleucel, have been approved for use in patients, and the approval of a third product, lisocabtagene maraleucel, is expected in 2020. The commercial availability of the first two products has facilitated the development of real-world experience in treating relapsed or refractory aggressive B-cell lymphomas, shed light on anti-CD19 CAR T-cell products' feasibility in trial-ineligible patients, and raised the need for strategies to mitigate the adverse effects associated with anti-CD19 CAR T-cell therapy, such as cytokine release syndrome, neurotoxicity, and cytopenia. In addition, promising clinical data supporting the use of anti-CD19 CAR T-cell therapy in patients with indolent B-cell lymphomas or chronic lymphocytic leukemia have recently become available, breaking the paradigm that these conditions are not curable. Multiple clinical CAR T-cell therapy-based trials are ongoing. These include studies comparing CAR T-cell therapy to autologous stem cell transplantation or investigating their use at earlier stages of disease, novel combinations, and novel constructs. Here we provide a thorough review on the use of the anti-CD19 CAR T-cell products axicabtagene ciloleucel, tisagenlecleucel and lisocabtagene maraleucel in patients with indolent or aggressive B-cell lymphoma or with chronic lymphocytic leukemia, and present novel CAR T cell-based approaches currently under investigation in these disease settings.

## Introduction

B-cell non-Hodgkin lymphomas (NHLs) constitute the disease setting in which treatment with chimeric antigen receptor-modified (CAR) T cells is currently most advanced. CD19 has been identified as an ideal target for CAR T-cell therapy in the majority of B-cell malignancies. In fact, while it is an ubiquitously expressed protein in the B lymphocyte lineage, its expression is maintained in B cells that have undergone neoplastic transformation (1). Early studies exploring the safety and feasibility of CD19-directed CAR T-cell therapy have generally included heterogeneous patient populations [i.e., they have enrolled patients with aggressive B-cell lymphoma, indolent B-cell lymphoma or chronic lymphocytic leukemia (CLL) in the same study] (2–4). However, later clinical trials focused mainly on aggressive B-cell lymphomas in light of their worse prognosis and higher need for novel curative options. For this reason, data regarding the efficacy of CAR T-cell therapy are more mature in the aggressive B-cell lymphoma setting, and the currently approved treatment indications exclude indolent lymphomas and CLL.

As of February 2020, two second-generation anti-CD19 CAR T-cell products, axicabtagene ciloleucel (axi-cel) and tisagenlecleucel (tisa-cel, formerly known as CTL019), have been approved by the U.S. Food and Drug Administration (FDA) and European Medicine Agency. Approval for a third product, lisocabtagene maraleucel (liso-cel [or JCAR017]), is expected in 2020. Alternative strategies aimed at improving CAR T-cell activity, reducing their toxicity, and expanding their indication to diseases other than aggressive lymphomas are undergoing clinical testing (Table 1).

**Table 1:** Ongoing trials of axi-cel, tisa-cel and liso-cel in patients with aggressive B-cell lymphoma.

**Trial**	**Phase**	**Patient population**	**Product**
NCT02348216 (ZUMA-1)	1/2	Failed 2 lines	Axi-cel
NCT02926833 (ZUMA-6)	1/2	Failed 2 lines	Axi-cel + atezolizumab
NCT03391466 (ZUMA-7)	3	Failed 1 line	Axi-cel vs ASCT
NCT03153462 (ZUMA-9)	EA	Failed 2 lines	Axi-cel (suboptimal)
NCT03704298 (ZUMA-11)	1/2	Failed 2 lines	Axi-cel + utomilumab
NCT03761056 (ZUMA-12)	2	Previously untreated	Axi-cel
NCT04002401 (ZUMA-14)	2	Failed 2 lines	Axi-cel + rituximab Axi-cel + lenalidomide
NCT04134117	1	PCNSL	Tisa-cel
NCT03630159 (PORTIA)	1	Failed 2 lines	Tisa-cel + pembrolizumab
NCT03876028	1	Failed 2 lines	Tisa-cel + ibrutinib
NCT04161118 (TIGER)	2	Failed 1 line (elderly)	Tisa-cel
NCT02445248 (JULIET)	2	Failed 2 lines	Tisa-cel
NCT03570892 (BELINDA)	3	Failed 1 line	Tisa-cel vs ASCT
NCT04094311	3B	Failed 2 lines	Tisa-cel (suboptimal)
NCT02631044 (TRANSCEND)	1	Failed 2 lines	Liso-cel
NCT03310619 (PLATFORM)	1/2	Failed 2 lines	Liso-cel + CC-122; Liso-cel + durvalumab
NCT03744676 (OUTREACH)	2	Failed 2 lines	Liso-cel
NCT03483103 (PILOT)	2	Failed 1 line	Liso-cel
NCT03575351 (TRANSFORM)	3	Failed 1 line	Liso-cel vs ASCT

*ASCT, autologous SCT; EA, expanded access; PCNSL, primary central nervous system lymphoma*.

Here we provide a detailed review of clinical trials and real-world experiences focused on the use of axi-cel, tisa-cel and liso-cel in patients with aggressive or indolent B-cell lymphomas or with CLL, also mentioning novel CAR T cell-based approaches currently under investigation in these disease settings.

## Axi-Cel for Aggressive B-Cell Lymphoma

### Efficacy and Predictors of Outcome

The safety and efficacy of axi-cel for the treatment of aggressive B-cell lymphoma was investigated in a pivotal, multicenter phase 1/2 study named ZUMA-1. The study included patients with refractory disease, defined as (1) progressive or stable disease as best response to the most recent chemotherapy regimen, or (2) progression/relapse within 12 months after autologous stem cell transplantation (SCT) (5, 6). Patients with diffuse large B-cell lymphoma (DLBCL), primary mediastinal B-cell lymphoma (PMBCL), or transformed follicular lymphoma (tFL) were eligible, whereas patients who had received allogeneic SCT or with prior and/or active central nervous system (CNS) disease were excluded.

In ZUMA-1, bridging therapy was not allowed. Conditioning consisted of fludarabine (30 mg/m^2^) and cyclophosphamide (500 mg/m^2^) administered intravenously for 3 days. At the most recent update published in 2019, 108 patients had been infused with a target dose of 2 × 10^6^ CAR T cells per kilogram of body weight (7). After a median follow-up time of 27 months, the best overall response rate (ORR) was 83% and the complete remission (CR) rate was 58%. Response was independent of traditional baseline prognostic factors except for the baseline high tumor burden, which was indicated by either an elevated lactate dehydrogenase (LDH) level or high total metabolic tumor volume detected on a positron emission tomography (PET) scan (8). The use of axi-cel resulted in 11.5-fold higher odds of CR and a 73% reduction in the risk of death when compared with standard salvage regimens (9).

Based on the results of this pivotal trial, axi-cel was approved by the FDA in 2017 for the treatment of relapsed or refractory large B-cell lymphoma in patients who had experienced failure of at least two lines of systemic therapy. Axi-cel is marketed by Kite Pharma (Santa Monica, CA) as Yescarta.

Researchers have made multiple efforts to identify product- and host-related factors predicting axi-cel's efficacy. To this end, the phenotype of the CAR T cells infused into 62 patients treated in the ZUMA-1 trial has been characterized (10). Axi-cel contains both CCR7+ T cells (naïve and central memory T cells) and more differentiated CCR7– T cells. The CCR7+:CCR7– T-cell ratio was positively associated with CAR T-cell peak and cumulative expansion during the first month after infusion. More robust CAR T-cell expansion was also associated with a higher likelihood of achieving CR (11), and early expansion of a specific subset of CD4+ CAR T cells (i.e., with high expression of CD45RO, CD57, PD1, and T-bet transcription factor) may help identify patients most likely to maintain CR at 6 months (12).

Pretreatment features of the host tumor microenvironment can affect the efficacy of axi-cel, and systemic inflammatory myeloid cytokines and circulating myeloid derived suppressor cells are associated with a higher risk of relapse after axi-cel therapy (13). On the other hand, CAR T cells themselves can influence the host tumor microenvironment. Both gene-expression profiling and multiple immunofluorescence analyses of paired pretreatment and posttreatment biopsy samples obtained from patients treated with axi-cel have shown upregulation of T-cell activation, effector, chemokine and immune-checkpoint genes (14, 15). Upregulation of immune-checkpoint genes has provided the rationale for combining axi-cel with checkpoint-inhibitor blockers for treatment of B-cell lymphoproliferative diseases (see below).

Of interest, in the ZUMA-1 trial, one-third of patients in partial remission (PR) 30 days after infusion had CR upon re-evaluation at day 90, and CR at day 90 was associated with prolonged survival (6). Of all PET scan-related measurements, only the total tumor glycolysis level at day 30 predicted the quality of response at day 90 (16). In addition, even in the absence of disease detected on PET scans, circulating tumor DNA could be detected as early as 28 days after axi-cel infusion and was associated with a poor outcome (17, 18). Novel techniques, aimed at the early identification of minimal residual disease (MRD) may guide the design of future consolidation studies and improve the outcomes in patients with aggressive B-cell lymphomas treated with axi-cel.

### Special Scenarios: Patients With Aggressive B-Cell Lymphoma Who Are Elderly or Have CNS Disease or Chronic Viral Hepatitis

Special scenarios that can be encountered during the use of axi-cel for the treatment of aggressive B-cell lymphoma include the presence of CNS disease or chronic viral hepatitis, or the need to treat it in elderly individuals.

Patients with CNS disease were excluded from the ZUMA-1 trial, so experience in regard to their treatment comes from the commercial use of axi-cel. A recent report from the U.S. Lymphoma CAR T Consortium on patients with aggressive B-cell lymphoma treated with axi-cel identified 17 patients with secondary lymphomatous CNS involvement (five had active disease at the time of infusion) and 283 patients without CNS disease (19). After a median follow-up of 10 months, similar rates of toxicity and comparable outcomes were observed in the two groups, highlighting the need to further explore the activity and safety of axi-cel in patients with B-cell lymphoma and CNS disease.

The ZUMA-1 trial also excluded patients with chronic viral hepatitis. In a recent case series, two patients with chronic hepatitis B and one with chronic hepatitis C were given commercial axi-cel (20). All three patients experienced a favorable outcome; viral reactivation was reported in only one patient who decided to interrupt anti-viral prophylaxis against medical advice. Currently, no conclusions can be drawn based on only three patients, and the safety of the use of this product will need to be further investigated in individuals with chronic viral hepatitis treated outside of clinical trials.

In spite of the absence of an age limit in the ZUMA-1 trial, only 24% of the included patients were older than 65 years, raising doubts about the safety and efficacy of axi-cel in this population. This concern is also supported by the notion that comorbidities, the burden of which is typically higher in elderly patients than in younger patients, are associated with poor outcomes after axi-cel infusion (21). Two small, retrospective single-center studies that analyzed outcomes for patients 65 years or older (24 and 17 patients, respectively, in the two studies) and patients younger than 65 years (25 and 44 patients, respectively) who received axi-cel for relapsed or refractory large B-cell lymphoma showed comparable efficacies and rates of cytokine release syndrome (CRS) and neurotoxicity (22, 23). Similar data were reported in a retrospective analysis of 177 Medicare patients (24). However, real-world multicenter data from a study comparing the outcomes of 153 patients 65 years or older with those of 244 patients younger than 65 years after treatment with axi-cel showed higher rates of neurotoxicity and atrial fibrillation in the older patients, although the higher atrial fibrillation rate was not associated with an increased mortality rate (25). Whereas older age and comorbidities should not preclude access to axi-cel, patients aged 65 years or older and those with comorbidities must be monitored closely and be the focus of experimental strategies aimed at mitigating toxicities.

### CRS, Neurotoxicity, and Persistent Cytopenia

In the ZUMA-1 trial, grade 3 or worse CRS occurred in 12 (11%) patients [as per the American Society for Transplantation and Cellular Therapy consensus criteria, the so-called Lee criteria (26)], and grade 3 or worse neurological events occurred in 35 (32%) patients (as per the Common Terminology Criteria for Adverse Events, version 4.03). Forty-seven percent of the patients received tocilizumab and 27% received steroids for the management of these side effects.

The U.S. Lymphoma CAR T Consortium recently reported that, in a large cohort of patients with aggressive B-cell lymphoma receiving axi-cel, those who did not have CRS were less likely to achieve CR, possibly reflecting lower short-term CAR T-cell activity, although no differences in other relevant outcomes, including the ORR and survival rate, were observed (27).

Fever is a defining characteristic of CRS, but its pattern and management remain elusive, frequently representing a challenge for the treating clinician. In a retrospective single-center study, fever occurred in 71% of patients within 72 h of axi-cel infusion, and it recurred in 69% of them after tocilizumab use (within 24 h in one-third of the cases) (28). As a result, more sensitive and specific tools for the early detection of CRS, including sublingual microcirculatory imaging, are currently under investigation (29).

Although the characteristics of CRS have been extensively described, CRS's clinical and radiological correlations with neurotoxicity were reported only recently (9). In a retrospective single-center study including 95 patients with relapsed or refractory large B-cell lymphoma treated with axi-cel, 65 patients experienced neurotoxicity. Among 26 patients with evaluable magnetic resonance imaging scans of the brain, 15 had abnormal findings, including autoimmune, encephalitis-like patterns in 6 patients; stroke-like patterns in 4 patients; leptomeningeal disease-like patterns in 3 patients; and posterior, reversible encephalopathy syndrome-like patterns in 2 patients. Electroencephalograms were performed in 52 patients and were abnormal for 47 of them. In 35 patients, focal and/or diffuse slowing, reported as an isolated finding, was the most common abnormality, whereas in 12 patients, epileptiform discharges and/or non-convulsive status epilepticus were observed.

Given the frequency and morbidity of neurotoxicity, multiple efforts are ongoing to recognize possible predictive factors. Among them, elevated fibrinogen levels on day 0, the germinal center B-cell subtype in DLBCL, and increased cortical glycolysis revealed on PET scans have already been identified (30, 31).

In patients treated with anti-CD19 CAR T cells, increasing evidence of the correlation between pre-treatment and post-treatment activation of the immune microenvironment and endothelial system with higher risk of both CRS and neurotoxicity has led to early use of steroids with the aim of preventing toxicity (32–34). In 41 patients treated in cohort 4 of the ZUMA-1 trial, steroid use was allowed in case of grade 1 neurotoxicity or grade 1 CRS after 3 days of supportive care (35). At a median follow-up of 9 months, a lower incidence of toxicity was observed in patients in cohort 4 who were supported with an earlier steroid use, in the absence of a significant impact on treatment efficacy. It is important to note that *post-hoc* analysis based on the cumulative dose and duration of steroid administration was not performed in this study, and given their potentially negative impact on CAR T cells efficacy, liberal use of steroids should not be encouraged.

A novel CAR T-cell-related toxic effect only recently fully acknowledged is persistent cytopenia, which has been observed in 30–40% of patients given axi-cel (6, 36). Persistent cytopenia is characterized by an increased need for blood or platelet transfusions, or the use of growth factors, the latter of which potentially increases the risk of CRS, with microscopic evidence of bone marrow failure without concomitant myelodysplastic syndrome in the majority of patients (37–40). Whereas myelosuppression resolves in 75% of individuals in the first year after axi-cel infusion, up to one-quarter of patients have persistently low numbers of CD4+ T cells 2 years after CAR T-cell infusion, with an increased risk of late opportunistic infections and a greater need for appropriate anti-microbial prophylaxis (41). In these patients, understanding the mechanisms of cytopenia, which is frequently unrelated to previous therapies and/or lymphodepleting conditioning, will be crucial for the effective prevention and management of this condition.

CAR T-cell-associated toxicity is frequently seen with the use of axi-cel in patients with aggressive B-cell lymphoma: the non-progression-related mortality rate is estimated to be 3–15% (42, 43), and it can constitute a significant financial burden for the health care system (44, 45). However, multiple studies have shown that the latter is overcome by the life-prolonging effects of treatment with axi-cel, resulting in an overall favorable cost-effectiveness balance (46, 47).

### Real-World Data on Axi-Cel's Efficacy and Safety

Since axi-cel's commercialization in 2017, the amount of real-world data regarding its efficacy and safety for the treatment of relapsed or refractory aggressive B-cell lymphoma has progressively grown.

In a recently updated retrospective multicenter analysis including 163 patients treated with axi-cel, at day 30 the ORR was 72% and the CR rate was 43%, both slightly lower than those observed in the ZUMA-1 trial, although researchers in the latter trial reported the best response to treatment instead of the 30-day response (48, 49). Grade 3-4 CRS was seen in 13% of patients and grade 3-4 neurotoxicity was seen in 41% of them. Whereas neurotoxicity was more frequent than that reported in the ZUMA-1 trial (31%), it is important to note that multiple scales, based on institution preference, were used for toxicity grading in this study. A post-marketing review of 397 case reports of neurotoxicity from the FDA Adverse Events Reporting System database has shown that the real-world neurotoxicity rates in patients given axi-cel largely resemble those reported in clinical trials. Specifically, neurotoxicity is more common in older patients and in those with concomitant CRS, and it is transient in the majority of cases according to patient-reported outcomes (50, 51). Similar data have been reported for 295 patients by the Center for International Blood and Marrow Transplant Research and for 122 patients by the National Health Service England, supporting the post-marketing efficacy and safety of axi-cel in both in the U.S. and Europe (52, 53).

While the excitement about the use of axi-cel in patients with relapsed or refractory aggressive B-cell lymphomas is rapidly growing, an important point to remember is that up to one third of the individuals for whom axi-cel was intended have been unable to receive it, mostly because of progression-related death before leukapheresis (54). This raises concern about the identification of optimal bridging therapy for these patients, as these strategies were not allowed in the ZUMA-1 trial and are typically needed in patients with high-risk disease (55). Unfortunately, real-world data show a heterogeneous pattern of the use of bridging therapies, and a preferred strategy has yet to be identified (56, 57). Of interest, recent data demonstrated that radiation therapy may be a safe and effective bridging approach; in particular, it may limit myelosuppression and favorably impact the host immune tumor microenvironment (58–60).

Finally, real-world data have shed light on the outcomes in patients who relapse after axi-cel treatment. In the U.S. Lymphoma CAR T Consortium's analysis of 274 patients with aggressive B-cell lymphoma who received axi-cel, 116 had disease progression during the follow-up period (maximum follow-up time, 14 months) (61). While patients whose disease progressed within 3 months had dismal outcomes, the effect of salvage therapy was more promising for those with a later relapse. To this regard, late progression represents a potential opportunity for further manipulation of CAR T-cell function and/or the host immune microenvironment, to achieve long term remission. Research efforts are undergoing to investigate these types of intervention.

## Tisa-Cel for Aggressive B-Cell Lymphoma

### Efficacy and Predictors of Outcome

Based on the promising results observed in 28 patients included in a single-center phase 2a study of tisa-cel (4), the efficacy of this drug was further investigated in a pivotal international, multicenter phase 2 trial named the JULIET study (36). The study included patients with aggressive B-cell lymphoma who had relapsed after at least two lines of systemic therapy, including an anthracycline-based regimen and an anti-CD20 monoclonal antibody, and who were either ineligible for or had disease refractory to autologous SCT. Patients with DLBCL; high-grade B-cell lymphoma, including double- and triple-hit lymphoma; or tFL were included in the study. Patients with PMBCL, who had received allogeneic SCT, or who had prior and/or active CNS disease were excluded.

In the JULIET study, bridging therapy was allowed, and conditioning therapy, consisting of either (1) fludarabine (25 mg/m^2^) and cyclophosphamide (250 mg/m^2^) given intravenously for 3 days, or (2) bendamustine (90 mg/m^2^) given intravenously for 2 days, was not mandated in patients with fewer than 1 × 10^9^ white blood cells within the week before tisa-cel infusion. In a first report, 111 patients were infused, including 95 in the U.S. (the main cohort) and 16 in Europe (cohort A). Ninety-three patients from the main cohort who had at least 3 months of follow-up were included in the efficacy analysis, and the best ORR was 52% (including 40% with CR); these results met the study's primary endpoint. Based on the results of this study, in 2018 the FDA approved tisa-cel for the treatment of relapsed or refractory large B-cell lymphoma in patients who had experienced failure of at least two lines of systemic therapy. Tisa-cel is marketed by Novartis (Basel, Switzerland) as Kymriah.

Updated efficacy data for 99 patients from the main cohort have been reported at a median follow-up of 19.3 months (62). The ORR was 54%, the CR rate was 40%, and the median duration of response was not achieved. Multiple efforts have been made to identify the factors associated with increased or impaired tisa-cel product efficacy in these patients. Factors examined have included clinical and laboratory baseline characteristics, and the use and type of bridging therapy and lymphodepleting conditioning. When considering the 115 patients from both cohorts who were infused with tisa-cel and underwent follow-up for at least 3 months, the only baseline characteristic associated with a lower ORR in multivariate analysis was a high pre-infusion LDH level (defined as higher than twice the upper limit of normal), which is a known surrogate marker of disease burden and aggressivity (63). Of these patients, the ORR for the 11 who did not receive bridging therapy was higher than that of patients who did receive bridging therapy (82% vs. 49%), likely reflecting a lower tumor burden and less-aggressive disease in the former group (64). Because of pre-infusional leukopenia, eight patients did not receive lymphodepleting conditioning and experienced a lower ORR (25%) than did those who received conditioning with fludarabine and cyclophosphamide (58%) or bendamustine (41%). These results highlight the importance of lymphodepletion in patients receiving tisa-cel (64).

The association between CAR T-cell persistence and efficacy also has been evaluated. In a subgroup analysis that included 93 patients from the JULIET trial, transgene levels in peripheral blood (measured using quantitative polymerase chain reaction) were equally detectable in tisa-cel responders and non-responders. The two groups had similar median times to maximum levels of circulating CAR T cells (9–10 days) and high inter-individual variability. In addition, whereas the majority of the responders had persistent transgene levels, some remained disease-free despite experiencing a decline in transgene amount to undetectable levels, demonstrating that quantitative polymerase chain reaction testing for CAR transgene levels in the blood is not a reliable basis for making any therapeutic decisions for patients with large B-cell lymphoma treated with tisa-cel (65).

### Special Scenarios: CNS Disease, Unmeasurable Disease, and Suboptimal Product Quantities

Special scenarios that can be encountered during the use of tisa-cel for the treatment of aggressive B-cell lymphoma include the presence of CNS disease, the absence of measurable disease before infusion, and the use of suboptimal quantities of product.

The outcomes for eight patients with active secondary CNS involvement at the time of commercial tisa-cel infusion have been reported (66). Three patients had parenchymal disease, three had lepto-meningeal disease, and two had both. Of these patients, four had responses (durable in three), and no patient experienced high-grade neurotoxicity. Of interest, although only two patients had concomitant active systemic disease at the time of infusion, CAR T-cell expansion (determined using patients' CD3 T-cell counts as surrogate marker) occurred in all patients, suggesting adequate trafficking of effector T cells to the CNS.

In the JULIET study, seven patients experienced CR with bridging therapy, and no measurable disease was observed at the time of tisa-cel infusion (67, 68). All seven patients remained in CR at 3 months and had toxicity grades and rates similar to those in the general study. Two patients had relapses by 1 year. Quantitative polymerase chain reaction-based assessment of CAR transgene levels showed adequate CAR T-cell expansion in all patients during the first 28 days after infusion, with a median time to peak expansion of 9 days, measurable levels of CAR transgenes at up to 2 years in the majority of patients, and a median time to undetectable CAR transgene levels of about 1 year. Collectively, these data support the efficacy of tisa-cel in patients without detectable disease and have potential implications for tisa-cel's use in a consolidative setting.

Prior to its release for commercial use, tisa-cel has to meet precise lot release specifications, such as the presence of 0.6 to 6.0 × 10^8^ viable CAR-positive T cells and a total cell viability of at least 80%. CAR T-cell products that do not meet these specifications can only be administered via an expanded access protocol or as single-patient investigational new drugs. Of interest, in the JULIET trial, where the median dose of viable CAR-positive T cells was 3 × 10^8^ (range, 0.1–6 × 10^8^ cells) and the transduction efficiency (as determined using flow cytometry) was 28% (range, 5–63%), CAR T-cell product attributes had no significant impact on tisa-cel's efficacy or toxicity (69). Likewise, the outcomes for 11 patients with refractory large B-cell lymphoma treated with “out-of-specification” tisa-cel have been analyzed, with efficacy and toxicity similar to those reported in the JULIET trial (70). These findings suggested that suboptimal tisa-cel products are safe and effective and that criteria for their commercial release may need to be revised in the future.

### CRS and Neurotoxicity

In the JULIET study, CRS was graded according to the University of Pennsylvania grading scale (71), and neurotoxicity was graded according to the Common Terminology Criteria for Adverse Events, version 4.03. Including all 111 patients, CRS of any grade was reported in 58% of the patients (grade 3-4 in 22%), and neurological events were reported in 21% of them (grade 3-4 in 12%). These conditions necessitated steroid and tocilizumab use in 10% and 14% of patients, respectively (36). With extended follow-up, which included 115 patients, the grade 3-4 CRS rate was 23%, and the grade 3-4 neurotoxicity rate was 11% (62). To ensure harmonization of grading among studies, CRS events in the pivotal JULIET study were recently regraded using the Lee criteria, showing a similar rate of CRS of any grade (57%), and a lower rate of grade 3-4 CRS (17%) (72). Similarly, after regrading using the CARTOX system (73), neurotoxicity of any grade was observed in 17% of patients and grade 3-4 neurotoxicity was observed in 13% (74).

In a 24-month clinical update of the JULIET study, increased serum C-reactive protein, ferritin, interferon-ɤ, interleukin (IL)-2, IL-6, and IL-10 levels after tisa-cel infusion were associated with severe CRS, with an increase as early as 2 days after infusion for IL-2, IL-6, and interferon-ɤ. In contrast, a weaker association with severe neurotoxicity was reported (75). Of interest, when analyzing the association between tisa-cel product characteristics and clinical outcomes, the total number of activated CD4+ T cells infused positively correlated with high-grade CRS but not with efficacy, suggesting that controlling the composition of the product helps mitigate toxicity without hampering efficacy (69).

### Real-World Data

The outcomes for 47 patients with relapsed or refractory large B-cell lymphoma treated with commercial tisa-cel were recently reported by the Center for International Blood and Marrow Transplant Research Cellular Therapy Registry (76). The ORR in these patients was 60% and the CR rate was 38%. Using the Lee criteria, grade 3-4 CRS was observed in 4% of patients and prompted steroids or tocilizumab use in 9 and 41% of patients, respectively. Grade 3-4 neurotoxicity was reported in 4.3% of patients, and no deaths were toxicity-related; all 14 observed deaths were secondary to progressive disease. Of interest, 21 of the 47 patients received “out-of-specification” commercial product, with no significant impact on efficacy or toxicity as compared with patients who received commercial tisa-cel within specifications. Similar results have been reported for a multicenter retrospective study, which described the outcomes of 79 patients with refractory large B-cell lymphoma treated with commercial tisa-cel at eight U.S. academic centers (49). In these patients, the ORR was 59% and the CR rate was 44%. Using institution-specific grading systems (including the Lee criteria, Common Terminology Criteria for Adverse Events [version 4.03], and CARTOX system), the grade 3-4 CRS rate was 1%, the grade 3-4 neurotoxicity rate was 3%, and use of steroids or tocilizumab was required in 7% and 13% of patients, respectively. Overall, these data suggest that, in the commercial setting, tisa-cel is safe and effective for patients potentially not meeting clinical trial eligibility criteria, although the management of these patients requires the expertise and resources that only academic centers can provide. The unexpectedly lower rates of CRS and neurotoxicity may reflect inconsistency in toxicity reporting outside of clinical trials, highlighting the importance of training healthcare professionals and of maintaining adequate electronic record systems.

## Liso-Cel for Aggressive B-Cell Lymphomas

### Efficacy, Predictors of Response, and Quality of Life

Liso-cel is a CD19-directed CAR T-cell product administered at a precise dose of CD4+ and CD8+ CAR T cells. The specific CD4+:CD8+ ratio is a defining characteristic of this product, and Celgene (Summit, NJ), the manufacturer of liso-cel, has made multiple efforts to reduce phenotype inter-patient variability and to decrease the risk of transducing residual tumor cells (77, 78).

The safety and efficacy of liso-cel are being investigated in a pivotal multicenter, seamless-design, phase 1 trial named TRANSCEND (79). Patients with large B-cell lymphoma that relapsed or was refractory after at least two lines of systemic therapy were eligible for the study. Patients with DLBCL, high-grade B-cell lymphoma (including double- and triple-hit subtypes), PMBCL and grade 3B follicular lymphoma (FL) were included in the study. Of note, patients with secondary CNS involvement were not excluded.

Bridging therapy was allowed, although patients without measurable disease before lymphodepleting conditioning with fludarabine and cyclophosphamide were excluded. Liso-cel was administered at one of three target dose levels (DLs): 50 × 10^6^ (DL1), 100 × 10^6^ (DL2), or 150 × 10^6^ (DL3) viable CAR T cells. DL2 was chosen for dose expansion. At the most recent update presented in December 2019, 268 patients had been infused (DL1, n=51; DL2, n=176; and DL3, n=41) and, because outcomes for the DLs have been similar, data have been pooled (80). Among the 255 patients evaluable for efficacy, at a median follow-up of 11 months, the best ORR was 73% and the CR rate was 53%, with a median duration of response of 13 months.

A phase 2 study, named OUTREACH, is now further investigating the efficacy of liso-cel at DL2 in the same population and will most likely lead to liso-cel's FDA approval and commercial availability in the near future.

In addition to efficacy data, the TRANSCEND study evaluated patient-reported outcomes to assess health-related quality of life, symptom burden, and health utility (81). The majority of patients experienced an improvement in health-related quality of life and health utility, and a reduction in their fatigue and pain burden up to 1 year after infusion, although some reported a temporary detriment during the first month after infusion. Like efficacy data, patient-reported outcomes will be crucial in the ongoing comparison of CAR T-cell products with autologous SCT.

Liso-cel is also being investigated in an open-label phase 2 study named PILOT as a second-line therapy for aggressive B-cell lymphoma in patients who are not eligible for transplant. Recently, preliminary efficacy data have been reported for the first nine patients: seven with large B-cell lymphoma and two with transformed lymphoma (82). At a median follow-up of 4 months, the best ORR was 100% and the CR rate was 44%.

As none of the traditional prognostic factors were significantly associated with efficacy in the TRANSCEND trial, to understand tumor microenvironmental factors affecting liso-cel clinical activity, multiplexed immunofluorescence was performed on biopsy samples obtained at baseline (*n* = 58) and on day 11 (*n* = 53; 28 paired) (83). As expected, early tumor infiltration by CAR and non-CAR T cells was associated with more durable responses. In addition, pre-treatment tumor infiltration by activated T cells and absence of macrophages were also associated with longer remissions, highlighting the importance of a functional and anti-tumoral microenvironment in increasing the success of CAR T-cell therapy. While potentially guiding the design of future combinations of CAR T cells with drugs with immunomodulatory activity, these data support the use of CAR T cells at earlier stages of disease and in less heavily pretreated patients.

### Special Scenario: CNS Disease

Unlike the JULIET and ZUMA-1 studies, the TRANSCEND study did not exclude patients with secondary CNS lymphomatous involvement. At the most recent update, nine patients with secondary CNS lymphoma have been treated with liso-cel: four at DL1 and five at DL2. Overall, three of the patients were given liso-cel as retreatment (84). Four patients responded, all of whom experienced CR, with two of these four patients having ongoing remission (at 270 and 545 days, respectively). Of interest, none of the three patients who received liso-cel as retreatment had responses. Only one patient had neurotoxicity (grade 3), which was successfully treated with steroids. Although details regarding concomitant systemic therapy, patient-specific CAR T-cell expansion, and CNS-directed bridging therapy have not been provided, patients with aggressive B-cell lymphoma who have CNS disease represent an unmet clinical need, and their inclusion in future clinical trials with CAR T cells should be strongly encouraged.

### CRS, Neurotoxicity, and Financial Burden

Among the 268 patients in the TRANSCEND study who were evaluable for safety, CRS of any grade (according to the Lee criteria) occurred in 42% of patients and grade 3-4 CRS occurred in only 2%. The median time of CRS onset was 5 days. Neurotoxicity of any grade (according to the Common Terminology Criteria for Adverse Events, version 4.03) was reported in 30% of patients and grade 3-4 neurotoxicity occurred in 10%. The median time of neurotoxicity onset was 9 days. Overall, treatment with tocilizumab or steroids were needed in 19 and 21% of patients, respectively (80). In the PILOT study, no patient had CRS or neurotoxicity and neither tocilizumab or steroids were used, but this may be accounted for by the limited follow-up and small sample size (82).

In light of liso-cel's unprecedentedly good safety profile, outpatient administration was allowed in the TRANSCEND and the PILOT studies. The outcomes of 37 patients given liso-cel in the outpatient setting were recently reported (85). Whereas 16 patients had CRS and 12 had neurotoxicity, only 2 had grade 3-4 complications, 3 needed tocilizumab with steroids, and 4 needed steroids alone. Overall, 22 patients needed hospitalization after a median of 5 days.

Although factors associated with safe outpatient administration of liso-cel have yet to be identified, if this product proves feasible for commercial outpatient use, the impact on the healthcare system may be considerable. A recent examination of health resource use among 102 patients treated with liso-cel in the TRANSCEND study showed that length of stay, either in a hospital or in an intensive care unit, is the key driver of toxicity management cost and is mainly associated with grade 3-4 toxicity (86). Likewise, an analysis of 94 patients from the same study showed that outpatient infusions of liso-cel were associated with a 40% shorter hospital/intensive care unit stay when compared with upfront inpatient infusions, entailing a reduction in the financial burden for patients and the healthcare system (87). Therefore, strategies aimed at mitigating toxicity in patients receiving CAR T-cell therapy, and allowing outpatient infusion and management are desperately needed.

## CD19-directed CAR T Cells for Treatment of Other B-Cell Lymphomas

Data regarding the use of CD19-directed CAR T-cell products in patients with indolent NHL [including FL and marginal zone lymphoma (MZL)] or mantle cell lymphoma (MCL)—the latter frequently representing an aggressive disease—are more scarce than those regarding the use of these products in patients with large B-cell lymphoma, and mainly derive from studies including patients with a variety of histologies. Based on the heterogeneity and small number of treated patients, and on the short follow-up periods in the majority of the reports, assessing the efficacy of these treatments with precision and identifying predictors of outcome or prognostic characteristics is difficult. Regardless, overall, the toxicity profile of CD19-directed CAR T-cell products in indolent NHL and MCL appears to be close to that observed in aggressive NHL cases. Larger studies specifically enrolling patients with indolent NHL and MCL are currently ongoing (Table 2).

**Table 2:** Ongoing trials of axi-cel, tisa-cel and liso-cel in patients with indolent B-cell lymphomas, MCL or CLL.

**Trial**	**Phase**	**Disease**	**Population**	**Product**
NCT02601313 (ZUMA-2)	2	MCL	Failed anthracycline or bendamustine, anti-CD20 mAb, and BTKi	Axi-cel
NCT04162756 (ZUMA-18)	EA	MCL	Failed anthracycline or bendamustine, anti-CD20 mAb and BTKi	Axi-cel
NCT03105336 (ZUMA-5)	2	MZL, FL	Failed 2 lines	Axi-cel
NCT03624036 (ZUMA-8)	1/2	CLL	Failed 2 lines (including BTKi)	Axi-cel
NCT03568461 (ELARA)	2	FL	Failed 1 line	Tisa-cel
NCT02631044 (TRANSCEND-NHL-001)	1	FL G3b, MCL	Failed 1-2 lines	Liso-cel
NCT03744676 (OUTREACH)	2	FL G3b	Failed 2 lines	Liso-cel
NCT03483103 (PILOT)	2	FL G3b	Failed 1 line	Liso-cel
NCT03310619 (PLATFORM)	1/2	FL G3b	Failed 2 lines	Liso-cel + CC-122Liso-cel + durvalumab
NCT03575351 (TRANSFORM)	3	FL G3b	Failed 1 line	Liso-cel vs ASCT
NCT03331198 (TRANSCEND-CLL-004)	1/2	CLL	Failed 2-3 lines	Liso-cel +/- ibrutinib

*BTKi, BTK inhibitor; EA, expanded access; G3b, grade 3b; mAb, monoclonal antibody*.

### Axi-Cel for Other B-Cell Lymphomas

Initial data on the efficacy of axi-cel for the treatment of indolent NHL were reported by the National Cancer Institute (88). Three patients with heavily pretreated FL and one with splenic MZL received cyclophosphamide (60 mg/kg) on days−6 and−7 and fludarabine (25 mg/m^2^) on days−5 through−1, followed by CAR T-cell infusion (0.3–3 × 10^7^ CAR T cells per kilogram of body weight), and a course of intravenous IL-2. Three patients had responses to the treatment, and a correlation between toxicity and serum-level elevations of inflammatory cytokines (i.e., interferon-γ and tumor necrosis factor α) was reported. After the treatment regimen was modified to omit IL-2 infusion, an additional patient with splenic MZL had a PR (2), and durable CR (>11 months) was reported in two patients with FL and one with MCL (89).

The phase II ZUMA-2 study is a registrational multicenter trial evaluating axi-cel in patients with relapsed/refractory MCL (90). Conditioning chemotherapy consists of cyclophosphamide (500 mg/m^2^/day) and fludarabine (30 mg/m^2^/day) for 3 days and is followed by a single infusion of axi-cel at a target dose of 2 × 10^6^ CAR T cells per kilogram of body weight. The most recent report includes data for 60 patients from the primary efficacy analysis, with a median of three prior treatment lines and a median follow-up period of 12.3 months. The ORR and CR rates were 93% and 67%, respectively, with a 12-month estimated progression-free survival rate of 61%. Grade 3-4 CRS (according to the Lee criteria) occurred in 15% of patients, and grade 3-4 neurological events occurred in 31% of patients, with no fatal events.

The ZUMA-5 phase 2, single-arm trial (NCT03105336), which is investigating the efficacy of axi-cel in patients with relapsed/refractory FL, MZL or other indolent NHLs, is currently enrolling, but results are not available yet.

### Tisa-Cel for Other B-Cell Lymphomas

Fourteen patients with FL that relapsed less than 2 years after the second line of therapy and who had a dismal prognosis (anticipated survival duration, <2 years) were treated with tisa-cel (4). Bridging therapy was allowed, and patients received variable lymphodepleting therapies, at the discretion of the treating physician, prior to the infusion of 1–5 × 10^8^ tisa-cel cells. The 3-month ORR was 79% and the 6-month CR rate was 71%, with 89% of patients who had a response remaining in remission at a median follow-up of 28.6 months. Two patients with MCL were also treated with the same regimen, and one of them had a response (91). At the most recent update presented in 2019, the median progression-free survival time in the FL patients was 32.4 months. At a median follow-up of 49 months, the median overall survival duration for the whole cohort as well as the median response duration for patients experiencing a CR were not reached (92).

The multicenter, phase 2 ELARA trial (NCT03568461) is currently enrolling patients with relapsed/refractory FL that will be treated with tisa-cel. Results are not available yet.

### Liso-Cel for Other B-Cell Lymphomas

A phase 1 study of JCAR014, a CAR T-cell product that uses the same construct as JCAR017 with the defined CD4+:CD8+ ratio, but includes an additional *in vitro* stimulation step with a CD19+ Epstein-Barr virus-immortalized lymphoblastoid cell line in the manufacturing process, enrolled 37 patients with relapsed/refractory B-cell NHL (3). Among five evaluable patients with FL, four had responses and two had a CR, whereas only one of four patients with MCL had a response.

Eight patients with FL and 13 with tFL were enrolled in a phase 1/2 clinical trial and given 2 × 10^6^ liso-cel per kilogram of body weight after cyclophosphamide- and fludarabine-containing lymphodepletion (93). Of the eight patients with FL, seven had received four or more prior lines of therapy, and four had experienced failure after autologous SCT. CR was achieved in seven of the eight patients at a median time of 29 days (range, 27–42 days), and was maintained in all patients at a median follow-up time of 24 months. Overall, the treatment was well-tolerated, and no significant differences in terms of toxicity between the FL and the tFL cohorts were observed. All-grade CRS and/or neurotoxicity were observed in 50% of patients with FL, and no adverse events of grade 3 or higher occurred.

Preliminary results for nine patients with relapsed/refractory MCL treated with liso-cel in the TRANSCEND study also have been reported (94). Six patients, with a median of five prior treatment lines, received 50 × 10^6^ CAR T cells (DL1) and three patients received 100 × 10^6^ CAR T cells (DL2). Three of the nine patients had CRS, all grade 1, and no neurological events occurred. The ORR was 78% (four of the six patients at DL1, at a median follow-up time of 12.4 months, and all three patients at DL2, at a median follow-up time of 1.4 month).

## CD19-directed CAR T Cells for CLL

In the initial pilot trials, CD19-directed CAR T cells were also evaluated in patients with relapsed/refractory CLL, but in this setting, CAR T-cell product development was slowed by a lower than expected ability to achieve CR, and a subsequently increased rate of progressive disease. Clinical studies designed specifically to assess the activity of anti-CD19 CAR T cells in CLL patients are underway (Table 2).

As in patients with indolent NHL, the timing and frequency of treatment-related complications in CLL patients receiving CAR T cells do not appear to be substantially different from those in patients with aggressive NHL. Also, due to a lower number of patients with CLL who have received CAR T cells, identifying disease-related predictors of response in these individuals is difficult. However, much translational work has been carried out and has revealed that the unsatisfactory efficacy of CAR T cells in CLL appears to be at least partially attributable to pre-existing immune system dysfunctions—particularly those affecting the T-cell compartment—which may limit the generation and expansion of effective autologous CAR T cells. Therefore, strategies to overcome this issue are currently under evaluation.

### Axi-Cel for CLL

The already-mentioned 2012 paper from the National Cancer Institute, describing the clinical results of patients treated with axi-cel and a course of intravenous IL-2, also included data on four patients with CLL who had previously undergone multiple relapses (88). Three patients had responses to axi-cel, one of whom had prolonged CR (>15 months). Four additional patients with CLL, who were treated with a modified treatment plan that did not include IL-2 infusion, had responses to axi-cel—including three who had CR (2).

The ZUMA-8 study (NCT03624036), which will specifically evaluate the safety and efficacy of axi-cel in patients with relapsed/refractory CLL, is currently recruiting patients.

### Tisa-Cel for CLL

Three patients with extensively pretreated CLL were initially enrolled in a pilot clinical trial and received tisa-cel. CAR T cells had a potent antitumor effect in all patients, two of whom experienced long-lasting CR (95). Thereafter, a total of 14 patients with relapsed/refractory CLL were successively treated during the course of the study (96). The patients received a variety of lymphodepleting regimens, followed by a median of 1.6 × 10^8^ tisa-cel cells infused over 3 days. The ORR was 57%, and 29% of the patients had CR with MRD negativity. Long-term persistence of tisa-cel was detected in patients who experienced CR, who had a median duration of response of 40 months. In contrast, the median duration of response in the four patients with PR was only 7 months. CR, PR and non-responding patients were also different in terms of tisa-cel peak expansion, which was significantly more robust in patients who had CR. CRS was observed in 9 of 14 patients and was grade 3-4 in 6 patients. Five patients had concomitant neurological symptoms and four received tocilizumab. CRS was also associated with peak expansion of tisa-cel (and with clinical response), and patients with grade 2-4 CRS had higher peak levels of IL-6 than did those with grade 0-1 CRS.

In addition, a dose-optimization trial was conducted to identify the optimal dose of tisa-cel in patients with CLL (97). Two doses (5 × 10^8^ and 5 × 10^7^ tisa-cel) were compared, and the results of the study suggested better efficacy of the higher dose (ORR, 55% vs. 31%; and CR rate, 36% vs. 8%). The optimal dose cohort was then expanded, and among 17 evaluable patients given 5 × 10^8^ tisa-cel, the ORR and CR rate were 53% and 35%, respectively. CR was maintained in five patients at a median follow-up duration of 23 months. CRS was experienced by 54% of patients and was of grade 3-4 in 20%. Interestingly, the tisa-cel dose was not associated with CRS development or severity.

### Liso-Cel for CLL

Thirteen patients with CLL were treated with JCAR014 in an initial phase 1/2 trial and had promising durable responses, with a CR rate of 50% (98). A cohort of 24 patients with CLL subsequently received the same product (99). These patients were particularly difficult to treat: they had received a median of five previous treatment lines, 19 had disease resistant to ibrutinib, and 6 had venetoclax-refractory disease. Of note, ibrutinib was held at the time of leukapheresis and CAR T-cell administration to avoid unexpected side effects. Lymphodepleting chemotherapy consisted of cyclophosphamide (60 mg/kg/day) and fludarabine (25 mg/m^2^/day) for the majority of patients, and after that patients received 2 × 10^5^ (*n* = 4), 2 × 10^6^ (*n* = 19), or 2 × 10^7^ (*n* = 1) CAR T cells. The ORR was 74% and the CR rate was 21%. Among evaluable patients who had bone marrow disease prior to CAR T-cell therapy, 88% had MRD negativity as determined using flow cytometry and 58% had MRD negativity as determined using deep immunoglobulin heavy chain sequencing. This deep response strongly correlated with outcome, and the patients who had CR and MRD negativity as determined via deep sequencing had 100% progression-free survival and overall survival rates (median follow-up time, 6.6 months). Twenty of 24 patients experienced CRS, which was grade 1-2 in 18 patients, grade 4 in one patient, and grade 5 in one patient (according to the Lee criteria). Two, five, and one patients experienced grade 1-2, grade 3, and grade 5 neurotoxicity, respectively. Overall, six patients needed the use of tocilizumab and/or steroids.

TRANSCEND-CLL-004 is an open-label phase 1/2 study of liso-cel in patients with relapsed/refractory CLL, which is currently recruiting. At the most recent update, results for 23 patients (22 of whom were evaluable for efficacy) were presented (100). After 3 days of lymphodepleting chemotherapy with fludarabine and cyclophosphamide, patients received 50 × 10^6^ (*n* = 9) or 100 × 10^6^ (*n* = 14) CAR T cells. In 83% of the patients CLL was defined as high-risk, and the patients had received a median of five prior therapies. All patients had previously received ibrutinib, and 21 of 23 were considered to have refractory disease or had relapses during therapy. At a median follow-up of 9 months, the best ORR was 82% and the best CR rate was 45%. By day 30, 60% of evaluable patients had undetectable MRD in the bone marrow. Of nine responding patients with a follow-up duration of 9 months or more, seven have remained progression-free, and in six patients responses deepened over time. Long-term CAR T-cell persistence was confirmed by the detection of transduced T cells at the 6-month timepoint in 80% of patients. CRS was reported in 74% of patients (grade 3-4 in 9%) and neurotoxicity was reported in 39% (grade 3-4 in 22%). Neurotoxicity was associated with a greater lymph node tumor burden and elevated levels of IL-6 or tumor necrosis factor α. Sixty-one percent of patients received tocilizumab and 48% received corticosteroids.

### Additional Anti-CD19 CAR T-Cell Products Under Evaluation in CLL

Another second-generation anti-CD19 CAR T-cell product has been developed and studied in patients with CLL by the group at Memorial Sloan Kettering Cancer Center. Similarly to that used for axi-cel, this construct includes a CD28 costimulatory domain and is delivered to T cells by a retroviral vector. The first report of the phase 1 trial included data on eight patients with purine analog-refractory CLL. Objective responses were not observed in either three patients who did not receive lymphodepleting chemotherapy prior to the infusion of 1–3 × 10^7^ CAR T cells per kilogram of body weight or in five patients who received cyclophosphamide (1.5 g/m^2^) prior to the infusion of 0.4–1.0 × 10^7^ CAR T cells per kilogram of body weight (101). More recently, after a modification of the protocol to optimize conditioning chemotherapy, the authors presented an updated report describing the outcome of a total of 16 patients who had received a median of four prior therapies (102). The ORR was 38% in the whole cohort and 50% in evaluable patients who had received conditioning chemotherapy (CR rate, 25%). All patients experienced CRS, which was generally an early event and precluded infusion of the second fraction of CAR T cells in 6 of 11 patients for whom split-dose treatment had been planned. Six patients experienced neurologic adverse events.

The same CAR T-cell product was used as consolidation therapy in eight patients who had residual CLL after receiving frontline chemoimmunotherapy with pentostatin, cyclophosphamide and rituximab (103). After low-dose conditioning therapy (cyclophosphamide, 600 mg/m^2^), escalating doses of CAR T cells were infused (3 × 10^6^, 1 × 10^7^, or 3 × 10^7^ CAR T cells/kg). This treatment approach appeared safe and well-tolerated, with no patients needing tocilizumab, corticosteroids or intensive care unit admission. Unfortunately, responses were observed in only 38% of the patients.

### CD19-directed CAR T Cells and Ibrutinib for CLL

Different studies have highlighted the impact of the T-cell subset distribution on final CAR T-cell products' antitumor efficacy. Besides the postulated improved potency of the already-mentioned optimized 1:1 CD4+:CD8+ T-cell ratio, which characterizes the liso-cel product, it was also shown in a mouse model that anti-CD19 CAR T cells derived from CD4+ and CD8+ naïve and central memory T cells are more effective than those derived from effector memory T cells (104). In patients with CLL, response to CAR T cells is associated with different parameters of T-cell fitness, and an elevated frequency of CD8+ T cells with memory-like characteristics (CD27+CD45RO–) in apheresis products contributes to clinical efficacy (105).

The Bruton tyrosine kinase (BTK) inhibitor ibrutinib has revolutionized the treatment landscape for CLL and has achieved unprecedented response durations also in patients with high-risk disease (106). Interestingly, in addition to its anti-neoplastic effect, ibrutinib exerts an off-tumor effect that modulates different immune compartments, and particularly T cells (107–109). The possibility of exploiting both ibrutinib's immunomodulatory activity and its antitumor effect to improve CAR T-cell performance is certainly very appealing.

Preclinical data have demonstrated that ibrutinib enhances intrinsic liso-cel activity *in vitro* and *in vivo* (110). Previous prolonged exposure of CLL patients to ibrutinib may favor tisa-cel expansion and CAR T-cell clinical activity, and in a mouse model, ibrutinib-based treatment improved CAR T-cell engraftment and cytotoxic efficacy (111). Also, pre-treatment with ibrutinib appears to modulate CAR T-cell phenotype, expanding CD8+CD62L+ (central memory) and shrinking CD62L– (effector/effector memory) T-cell subsets in the final CAR T-cell product (102, 112).

CTL119 (the humanized version of CTL019) was combined with ibrutinib to treat CLL in 19 patients who were not in CR despite at least 6 months of ibrutinib therapy (113). After standard lymphodepletion, patients received 1–5 × 10^8^ CTL119 cells over 3 days. Five patients were receiving first-line ibrutinib, and for the remaining 14, the median number of prior therapies was two. Three patients had experienced failure of prior treatment with murine CD19-directed CAR T cells without ibrutinib. The 3-month ORR for the entire cohort was 71%, with a CR rate of 43% and a bone marrow remission rate of 94% (including 78% MRD negative responses as determined via deep sequencing). At a median follow-up of 18.5 months, the majority of patients were still receiving ibrutinib (the median time from CAR T-cell infusion to ibrutinib discontinuation was 8 months; *n* = 6). CRS was common (95%), but was of grade 3-4 in only 16% of the patients. Five patients experienced encephalopathy (four grade 1-2, one grade 4), and one patient died of a cardiac arrhythmia that occurred during an episode of severe neurotoxicity.

A study performed at the Fred Hutchinson Cancer Research Center compared outcomes of CLL patients treated with JCAR014 (2 × 10^6^ CAR T cells/kg) in a phase 1/2 trial (*n* = 19) with those of a subsequent cohort in which JCAR014 was administered with concurrent ibrutinib from at least 2 weeks prior to leukapheresis until at least 3 months after CAR T-cell infusion (*n* = 17) (114). Although not statistically significant, the ORR in the ibrutinib cohort was superior to that in the non-ibrutinib cohort (88% vs. 56%). The rate of patients experiencing all-grade CRS was similar in the ibrutinib and non-ibrutinib cohorts (76% vs. 89%; *p* = 0.39), but in the former cohort, no patients had grade 3-5 CRS (vs. 26% of patients in the non-ibrutinib cohort). Among patients receiving ibrutinib, one died, presumably from a fatal cardiac arrhythmia, in the setting of grade 2 CRS. Ibrutinib did not appear to influence the frequency or severity of neurotoxicity or cytopenias.

Finally, in the already-mentioned cohort treated at Memorial Sloan Kettering Cancer Center, response of CLL to CAR T-cell therapy appeared better in the patients receiving ibrutinib concurrent with cellular therapy than in the patients who did not receive ibrutinib (4 of 5 vs. 2 of 11 responders), although the number of patients studied was small and the efficacy of the tested CAR T-cell product was suboptimal (102).

Overall, these initial data have demonstrated the safety of treatment with ibrutinib during T-cell collection and CAR T-cell infusion and, in spite of the short follow-up periods, they support the hypothesis that treatment with ibrutinib increases CAR T-cell antitumor activity. The exact mechanisms through which ibrutinib modulates CAR T-cell efficacy are still under investigation.

## Novel CAR T Cell-Based Approaches for B-Cell NHL and CLL

Undeniably, the commercial availability of CD19-directed CAR T cells is a real game-changer in the treatment landscape for aggressive B-cell NHL. Besides those already mentioned, other second-generation anti-CD19 products are under investigation (e.g., JWCAR029) (115). However, to improve the clinical activity of CAR T cells and expand the therapeutic indications for these treatments to disease settings in which results are still suboptimal, further research efforts are needed. Several approaches have been tested, and some of them are currently being evaluated in the clinical setting.

### Third-Generation CD19-Directed CAR T Cells

The co-stimulatory domain inserted into the CAR structure appears to influence the activity of the transduced effector cells. CD28 generates a more rapid and intense signal than does 4-1BB, but this leads to shorter periods of cytotoxic activity and decreased *in vivo* CAR T-cell persistence (116–118). With the aim of combining rapid tumor elimination and long-term durability in the same product, third-generation CAR T-cell constructs include two different co-stimulatory domains in their structures.

In a phase 1 trial, 16 patients with NHL were simultaneously infused with second-generation anti-CD19 CAR T cells containing the CD28 co-stimulatory sequences alone and with third-generation CAR T cells containing both CD28 and 4-1BB (119). Cells transduced with the third-generation vector had superior expansion and longer persistence, suggesting that the addition of 4-1BB enhances the *in vivo* kinetics of CD28-containing CD19-directed CAR T cells. This same construct (i.e., containing both CD28 and 4-1BB), developed at Baylor College of Medicine, was used in a phase 1/2a study: among 11 patients with relapsed/refractory CD19+ NHL or CLL, response rate was low (4 of 11 patients) and not very durable, although only a minority of the patients experienced CRS or neurological toxicity (120).

Investigators in a single-center phase 1/2 trial currently running in Germany are treating a broad spectrum of patients with CD19+ relapsed/refractory NHL with another third-generation anti-CD19 CAR T-cell product incorporating both CD28 and 4-1BB (121, 122). The preliminary data have demonstrated a very reassuring toxicity profile with no CRS or neurologic events of grades higher than 2, and good responses in the first eight patients treated (ORR, 75%).

### Alternative Targets and Dual Targeting

A possible approach to overcoming resistance to CD19-directed CAR T cells is to target a different antigen.

The established use of anti-CD20 monoclonal antibodies in B-cell NHL therapy supported the development of anti-CD20 CAR T cells. Different CD20-directed constructs have been tested by researchers at Fred Hutchinson Cancer Research Center, but they lacked optimization for successful *in vivo* use (123–125). Another second-generation anti-CD20 CAR T-cell product was used to treat relapsed/refractory DLBCL in seven patients. Among six evaluable patients, five had a response (mainly PRs) (126). In a subsequent phase 2a study, 11 patients with CD20+ NHL underwent treatment and had an ORR of 82%, a CR rate of 55%, and an acceptable toxicity profile (no grade 4 toxic effects or CRS events were reported) (127).

CD22 has been evaluated as a candidate target antigen mainly in B-cell acute lymphoblastic leukemia, but clinical trials including patients with refractory CD22+ NHL are ongoing (NCT02794961, NCT02315612).

Another attractive target may be the immunoglobulin kappa (κ) light chain, which allow to direct CAR T-cell cytotoxic activity toward κ chain-restricted B-cell lymphomas, avoiding complete B-cell aplasia. In a phase 1 trial, nine patients with relapsed or refractory NHL or CLL were treated with κ chain-directed CAR T cells. The ORR was 33% (CR, one patient; PR, two patients), and no significant treatment-induced toxic effects were observed (128).

The receptor tyrosine kinase-like orphan receptor (ROR1) is an embryonal antigen aberrantly expressed in some cancers, including CLL and MCL. Preclinical evidence and *in vivo* evaluations in non-human primates support the potential antitumor efficacy of a ROR1-directed CAR T-cell strategy (129, 130), but clinical results in humans are lacking.

In the absence of another widely and homogeneously expressed target that could replace CD19 in treatment of B-cell lymphoproliferative diseases, the concomitant targeting of more than one antigen was favored as an alternate strategy (dual CAR T cells). In acute lymphoblastic leukemia, targeting CD19 and CD123 may be effective for the treatment and prevention of CD19-negative relapses. In the preclinical setting, a CAR-expressing T-cell population targeting both CD19 and CD123 showed superior *in vivo* activity compared to that of anti-CD19 CAR T cells, anti-CD123 CAR T cells, and pooled anti-CD19 and anti-CD123 CAR T-cell populations (131).

For treatment of NHL, the selection of two clinically validated targets for dual CAR T cells, such as CD19 and CD20, is certainly very appealing (132). In a phase 1 trial, bispecific CD19/CD20 CAR T cells were used to treat relapsed/refractory B-cell NHL and CLL (133). All patients received lymphodepletion (fludarabine and cyclophosphamide) followed by CAR T cells (starting dose, 2.5 × 10^5^ cells/kg; target dose, 2.5 × 10^6^ cells/kg, which was the dose selected for the expansion phase). Among 11 treated patients (5 with DLBCL, 4 with MCL, and 2 with CLL), the ORR was 82%, the CR rate was 55%, and all CR patients were still in remission at the latest follow-up. Fifty-five percent of patients had CRS (all grade 1-2) and 27% had neurotoxicity (all grade 1-2).

A bispecific CAR construct targeting CD19 and CD22 was used in a phase 1 trial to treat DLBCL in five patients (134). One patient had CR and two had PR, with overall acceptable tolerability (CRS, 86% of patients; neurotoxicity, 43% of patients, all grade 1-2). An additional CAR T-cell product targeting CD19 and CD22 (AUTO) is currently under investigation in patients with DLBCL (NCT03287817).

Finally, the possibility of concomitantly targeting CD19 and CD37, an antigen expressed by B-cell NHL and CLL cells, has been explored preclinically (135).

### CAR T Cell-Based Combination Strategies for the Treatment of B-Cell NHL and CLL

Besides the already-mentioned combination of CAR T cells with ibrutinib in CLL patients, other therapeutic associations of CAR T cells and drugs that affect the immune system are under evaluation with the aim of improving T-cell fitness and, subsequently, the CAR T-cell efficacy.

When the programmed cell death protein 1 (PD-1)-blocking antibody pembrolizumab was administered to 12 patients with refractory B-cell NHL who experienced progression after anti-CD19 CAR T-cell therapy, it induced a CAR T-cell re-expansion peak in 75% of treated patients, and an antitumor response in 27% of them (136). The phase 1b PORTIA trial is currently investigating the safety and efficacy of tisa-cel plus pembrolizumab (200 mg given every 21 days for up to six doses) in patients with relapsed/refractory DLBCL. A preliminary report on four patients who received pembrolizumab starting on day 15 after tisa-cel administration showed no exacerbation or recurrence of CAR T cell-related toxic effects following pembrolizumab infusion, but also no secondary CAR T-cell expansion (137).

The ZUMA-6 phase 1 study is investigating axi-cel in combination with the anti-programmed death-ligand 1 (PD-L1) monoclonal antibody atezolizumab (1,200 mg given every 21 days for four doses, starting at different timepoints after CAR T-cell infusion) (138). Pharmacokinetic data suggested enhanced axi-cel expansion, and preliminary results for 12 patients have shown an ORR of 90%, a CR rate of 60%, and durable responses. No apparent exacerbation or recurrence of axi-cel-related toxic effects following atezolizumab infusion were noted.

In another phase 1 study, the combination of JCAR014 and the anti-PD-L1 monoclonal antibody durvalumab (administered in different doses and schedules) was evaluated in patients with relapsed/refractory aggressive B-cell NHL (139). In the preliminary report of 15 treated patients, *in vivo* re-expansion of CAR T cells was observed in a minority of patients, the ORR was 50%, and the CR rate was 42%. Thirty-eight percent of the patients had CRS (grade 1-2 in 31%) and 8% experienced grade 1 neurotoxicity.

The ongoing phase 1/2 PLATFORM trial is investigating the efficacy of liso-cel in association with different immune system modifiers — in parallel cohorts — in patients with aggressive B-cell NHL. Arm A of the trial is combining liso-cel with durvalumab (starting on day 29 at a total dose of 1,500 mg/4 weeks for up to 12 months) (140). Of the 11 patients who have received liso-cel and at least one dose of durvalumab, 10 have had responses, including 7 CR. No dose-limiting toxic effects were observed, and CRS did not occur after durvalumab infusion. Although the follow-up is still short and the number of treated patients is low, this trial suggests better persistence of CAR T cells over time when liso-cel is given with durvalumab instead of alone. This persistence may possibly convert into more durable responses.

Lenalidomide and other immunomodulatory drugs (IMIDs) can also be used to recover T-cell exhaustion. Preclinical data have demonstrated that lenalidomide enhances the anti-tumor activity and persistence of CAR T cells in different NHL and multiple myeloma models (141–143). Of note, the already mentioned PLATFORM trial also includes an arm in which patients will receive CC-122, a novel IMID, and the phase 1/2 ZUMA-11 trial (NCT03704298) is investigating the safety and efficacy of axi-cel in combination with utomilumab, a monoclonal antibody that binds 4-1BB and stimulates immune cells, in patients with refractory DLBCL.

Finally, it has been proposed that a further enhancement of CAR T-cell activity might be indirectly pursued through the manipulation of the gut microbiome, based on evidences supporting its critical role in the responses to other immune-based treatments, such as checkpoint inhibitors (144).

### Armored CAR T Cells

A further strategy to increase CAR T-cell efficacy envisages the induction of the expression on effector T cells—along with the CAR—of an additional transgene, which endows them with supplementary functions, such as enhanced effector activity, or a control over microenvironment-induced immunosuppression.

TRUCKs (T cells redirected for universal cytokine killing) are CAR T cells that produce and release a transgenic cytokine, that accumulates in the targeted tissue. The constitutive or inducible expression of IL-12, IL-15, and IL-18 has been integrated in different models of CAR T cells, demostrating benefical effects in terms of efficacy (145–147).

Based on the aforementioned efficacy of the combination of CAR T cells with checkpoint inhibitor antibodies, CAR T cells can also be engineered to secrete anti-PD-1 or anti-PD-L1 blockers directly at the site of the immune recognition (built-in CAR T-cells) (148).

Furthermore, armored CAR T cells can be modified to express ligands for costimulatory molecules. For example, in an acute lymphoblastic leukemia preclinical model, the expression of CD28-co-stimulated CAR along with 4-1BB-L increases the persistence and tumoricidal effect of transduced effector T cells, optimizing the engagement of both CD28 and 4-1BB signals (117). A phase 1 clinical trial evaluating escalating doses of autologous 19-28z/4-1BB-L+ CAR T cells (from 1 × 10^5^ to 3 × 10^6^ cells/kg) in patients with NHL or CLL is ongoing, and preliminary data have been presented (149). Of 27 patients who received treatment, 16 had CR, including 78% of patients with DLBCL, 75% of patients with FL, 33% of patients with CLL and 67% of patients with Richter's transformation. No dose-limiting toxic effects were observed. CRS occurred in 39% of patients (one patient had a grade 3 event), and neurotoxicity occurred in 39% of patients (three patients had a grade 3 event).

CAR T cells can also be modified to counteract immune-suppressive signals. In mouse models of solid tumors, CAR T cells expressing a chimeric construct comprising the extracellular domain of PD-1 and the cytoplasmic signaling domains of CD28 demonstrated improved antitumor activity associated with decreased susceptibility to tumor-induced hypofunction, and attenuation of inhibitory-receptor expression (150). Anti-CD19 CAR T cells co-expressing the PD-1/CD28 chimeric switch receptor were evaluated in a phase 1 clinical trial treating patients with relapsed/refractory DLBCL (151). In 17 patients, conditioning chemotherapy with cyclophosphamide and fludarabine was followed by CAR T-cell infusion at a dose of 0.5–4.0 × 10^6^ cells/kg. The ORR was 59% and the CR rate was 41%. Eighty-two percent of the patients had CRS (grade 1-2) and 24% had neurotoxicity (grade 1).

Instead of inducing the expression of an additional gene, a complementary approach is the silencing of the checkpoint inhibitor molecule PD-1 on CAR T cells, in order to affect the immunosuppressive tumor microenvironment at the site of effector T-cell activity (NCT03208556, NCT03298828).

### Allogeneic CAR T-Cell Products

The use of allogeneic anti-CD19 CAR T-cell products obtained from healthy donors (off-the-shelf products) could represent a solution to avoid the need to use patient-derived dysfunctional T cells, increase availability of the product without the need to wait for time-consuming manufacturing, and reduce costs, as allogeneic manufacturing can generate products for use in several patients. Importantly, however, strategies to minimize the alloreactivity of donor-derived T cells, determined by their T-cell receptor (TCR) reaction against non-autologous tissues, must be implemented before off-the-shelf products can be used. Allogeneic products could in fact cause graft-vs.-host disease, but also induce CAR T-cell rejection due to the recipient immune system acting against the infused cells.

Recently developed gene editing technologies have been fundamental to prevent the expression of endogenous TCR on modified T cells. Different techniques can be exploited in order to disrupt the TCR α constant (TRAC) gene, such as the use of zinc finger nucleases (ZFN), transcription activator-like effector nucleases (TALEN), and CRISPR/Cas9 system (152).

A universal anti-CD19 CAR T-cell product (UCART19) has been generated by simultaneously introducing the CAR, and knocking out the TCR (to avoid graft-vs.-host disease) and CD52 (to induce resistance to the anti-CD52 monoclonal antibody, used in lymphodepletion to reduce the risk of UCART19 rejection) in allogeneic T cells (153). UCART19 has been used to treat relapsed/refractory B-cell acute lymphoblastic leukemia; evidence of UCART19 expansion was seen in responding patients and safety profile was acceptable and manageable (154). ALLO-501, another allogeneic product with the same construct as UCART19, is currently under clinical investigation in the ALPHA clinical trial (NCT03939026) for the treatment of relapsed/refractory DLBCL and FL.

PBCAR0191 is an allogeneic anti-CD19 CAR T-cell product generated with a single-step CAR knock-in and TCR knock-out. It includes a novel costimulatory domain (N6) that promotes cell expansion while maintaining the naïve cell phenotype. In a phase 1 trial, preliminary evidence of *in vivo* cell expansion and of a cell-mediated antitumor effect was seen in three patients with NHL who received the initial dose level (3 × 10^5^ CAR T cells/kg) (155).

Finally, it is worth highlighting that a CAR construct may be transduced also into other immune cells, such as natural killer (NK) cells. Allogeneic NK cells represent an attractive carrier for CAR because they are efficient immune-effector cells and do not cause graft-vs.-host disease. CD19-directed CAR NK cells have been generated from cord blood; these cells, which also incorporate the IL-15 gene (to support NK cell survival and proliferation) and a so-called suicide gene (inducible caspase-9, to induce the selective elimination of transduced cells in case of excessive toxicity), have proven to be very effective in preclinical NHL and CLL models (156). A clinical trial performed at The University of Texas MD Anderson Cancer Center has evaluated the safety and efficacy of cord blood-derived, anti-CD19, CAR-engineered NK cells for the treatment of B-cell lymphoid malignancies (157). No CRS neither neurotoxicity were observed. Eleven patients were treated and 8 (73%) had a response, including CR in 7 (4 with lymphoma and 3 with CLL).

## Conclusions

CAR T-cell therapy is rapidly modifying the treatment and outcome of aggressive and indolent B-cell lymphomas and CLL, and is becoming established as a new therapeutic option. Ongoing clinical trials are further improving the efficacy and curative potential of CAR T cells, also investigating their rationally-designed combinations with drugs with immunomodulatory activity, and in the future, novel off-the-self products may facilitate more rapid and broadly-accessible treatments. Real-world data are increasingly demonstrating the feasibility of CAR T-cell therapy in patients otherwise ineligible for clinical trials, and supporting the need for novel strategies to mitigate CAR T-cell-related toxicity. Preclinical studies and patient sample-based analyses are urgently needed to clarify the biological mechanisms of such toxicities, and to enable outpatient infusion of the majority of CAR T-cell products, minimizing healthcare-associated costs.

## Author Contributions

CV and PS developed the idea, designed the review, and wrote the manuscript.

## Conflict of Interest

The authors declare that the research was conducted in the absence of any commercial or financial relationships that could be construed as a potential conflict of interest. The handling editor declared a shared affiliation, though no other collaboration, with one of the authors PS.
